# Effect of Silica Microparticles on Interactions in Mono- and Multicomponent Membranes

**DOI:** 10.3390/ijms232112822

**Published:** 2022-10-24

**Authors:** Beata Tim, Monika Rojewska, Krystyna Prochaska

**Affiliations:** 1 Faculty of Materials Engineering and Technical Physics, Poznan University of Technology, Ul. Piotrowo 3, 60-965 Poznan, Poland; 2 Institute of Chemical Technology and Engineering, Poznan University of Technology, Ul. Berdychowo 4, 60-965 Poznan, Poland

**Keywords:** cholesterol, silica microparticles (MPs), DPPC, Langmuir monolayer, π–A isotherm, the relaxation of phospholipid film

## Abstract

Advancing our understanding of the mechanism of the interaction between inhaled pollutant microparticles and cell membrane components is useful to study the impact of fine particulate matter on human health. In this paper, we focus on the effect of cholesterol (Chol) molecules on the surface properties of a model membrane in the presence of silica microparticles (MPs). Mixed monolayers containing phospholipid-dipalmitoylphosphatidylcholine (DPPC), Chol and silica particle dispersions (MPs; 0.033% *w/w*, 0.33% *w/w* and 0.83% *w/w*) were formed and studied using the Langmuir monolayer technique complemented by Brewster Angle Microscopy (BAM) images. It was shown that Chol caused a condensation of the DPPC monolayer, which influenced the penetration of MPs and their interactions with the model membrane. The relaxation experiments of the lipid–MP monolayer proved that the presence of Chol molecules in the monolayer led to the formation of lipid and MP complexes. Strong interactions between Chol and MPs contributed to the formation of more stable monolayers. The presented results can be useful to better comprehend the interaction between particulate materials and the lipid components of biomembranes.

## 1. Introduction

The unflagging popularity of materials containing both microparticles and nanoparticles is the reason for more and more research into the potential effects of their use. Despite the fact that there are many important and useful applications of such materials in various areas of life [1,2,3,4], it cannot be ignored that the effects of their impact on the environment and living organisms have not yet been fully understood.

The presence of microparticles in the air contributes significantly to the formation of smog, the presence of which in the atmosphere negatively affects the health of many people. This problem has become extremely popular in recent years, and the effect of particulate matter (PM) on living organisms has begun to be investigated. The respiratory system is the most common way for fine PM 2.5 (particle diameter ≤2.5 µm) and PM 10 (particle diameter ≤10 µm) to enter the body. As a result of inhalation, PMs enter the lungs, and when the concentration limit is exceeded, they can cause respiratory system diseases [5]. Additionally, solid particles, due to their size, also enter the body through the digestive system, skin and tear film. In general, human exposure to airborne microparticles depends on their size. The large particles (>10 µm) are assumed to accumulate in the upper airways upon respiration, whereas PM 10 can penetrate the bronchioles, and PM 2.5 and smaller may even penetrate the alveoli [6]. As can be assumed, the size of microparticles can affect their toxicity, because smaller particles are more favorable to interact with cells and tissues [7]. However, Dwivedi et al. [8] studied how the size of hydrophobic nanoparticles (NPs) impact lung surfactant model membranes at a molecular level. They proved that larger NPs are responsible for causing more serve structural and functional damage to the model lipids films (such as the dipalmitoylphosphatidylcholine (DPPC) monolayer). In the presence of larger NPs, the DPPC film was better compressible because the network structure of the model lipid film was disrupted by the diffusive mixing of rigid and fluid domains. Therefore, DPPC monolayers showed a decreased tendency for phase separation due to a reduction in the line tension. Moreover, the mechanism for the inhibitory effect based on the size of the NPs was also discussed. They showed that fewer numbers of larger NPs had much more structural and functional damaging effects on the DPPC monolayer than the comparatively higher number of smaller NPs. Moreover, Allegri et al. [9] also showed that the shape of the microparticle strongly impacts its toxicity by modifying interactions with cells and tissues. They showed that microfibers interact with cells and tissues differently than films, microspheres or fragments.

The fast development of global technology has been accompanied by a rapidly accumulating amount of waste in the environment, especially in the air. Atmospheric transport and many processes such as tire wear and abrasion from synthetic textiles produce a lot of microscopic particles, e.g., those containing plastic, wood or silica. Moreover, natural silica dust that comes from the Sahara is also associated with respiratory diseases and silicosis [10].

The microparticle pollutants that are most often associated with occupational diseases include: asbestos, silica dust, soot, wood dust, cotton dust and hay dust. In this paper, we set our focus on silica microparticles (MPs), which are characterized as airborne fragments usually in the breathable range <10 µm, mostly between 0.5 and 2 µm [11]. The inhalation of silica (silicon dioxide) dust is a well-known cause of occupational diseases, predominantly fibrotic lung disease (silicosis). Moreover, exposure to high concentrations of silica dust is also associated with chronic obstructive pulmonary disease, lung cancer, autoimmune diseases and renal disease. Those who are mainly exposed are workers in mining, brick working, construction and road repair. Inhaled silica particles accumulate in the airways and are internalized by macrophages. The uptake by the macrophages leads to the death of the cell. After that, the silica particles are released and intensify the inflammation of the organism [12].

With this in mind, many studies have been conducted to explain the mechanism of the impact of solid particles on the human body. In this approach, cell membranes play an important role since they constitute a barrier between the various structures of the body and the environment. The complex structure of biological membranes makes it possible to use mono- and multicomponent membranes in research. One of the biomimetic membrane systems used are lipid monolayers, which are often composed of DPPC, which is a component of the erythrocyte membrane or pulmonary surfactant. In addition, other lipids such as cholesterol (Chol) or proteins are also added to form multicomponent monolayers [13,14,15]. Monolayers are formed at the air–water interface, which allows for the acquisition of a lot of information about the interactions and the physicochemical properties of its components. To obtain stable lipid monolayers that simulate biological model membranes, the Langmuir method can be used, which is a good technique to study the structures and properties of lipids, proteins and surfactants [16,17,18]. Furthermore, the properties of model lipid monolayers are similar to those of real biological membranes [19,20]. Thanks to this, the Langmuir technique has found applications in many fields [20,21,22,23].

Many studies have been carried out regarding the effects of solid particles on both monolayers and multicomponent lipid monolayers [24,25,26]. Monitoring the influence of particles on monolayers allows one to observe the interactions occurring as a result of the interaction of components on model membranes and to determine changes in their phase states. In our previous work [27], we examined the effects of silica microparticles on biological model membranes consisting of DPPC and POPE (2-oleoyl-1-palmitoyl-*sn*-glycero-3-phosphoethanolamine). The phospholipids selected for the study were characterized by a different chemical structure, because the structure of the phospholipids and their composition in the membrane may affect the strength of chemical barriers [28]. Studies have shown that the presence of silica microparticles leads to a different phase behavior of the monolayer and also affects the morphology of the lipid membrane. In this work, we decided to extend this research and investigate the effects of silica microparticles (MPs) on membranes consisting of DPPC and Chol. Chol is also an important component of cell membranes. In the case of eukaryotic membranes, the number of Chol molecules can be up to two times greater than that of phospholipid molecules. In the bilayer, the Chol molecules are arranged so that their hydroxyl groups are near the phospholipid heads, and the hydrophobic rings and side chains in the fatty acid chains point towards the interior of the membrane. Furthermore, due to the interaction with other lipids, Chol significantly influences the fluidity of the membrane [29,30].

In the present investigation, MPs were introduced into the lipid monolayer. The effect of solid particles on the behavior of the DPPC or DPPC + Chol monolayer in real time was described based on the surface pressure isotherms (π) − area (A), compressibility modulus (*C_s_*^−1^) − surface pressure (π) and Brewster Angle Microscopy (BAM). In addition, relaxation experiments were performed to determine the effect of the concentration and distribution of MPs on the stability of model cell membranes.

## 2. Results and Discussion 

### 2.1. Impact of Cholesterol on the Model Lipid Monolayer with/without Silica Microparticles

**Figure 1:**
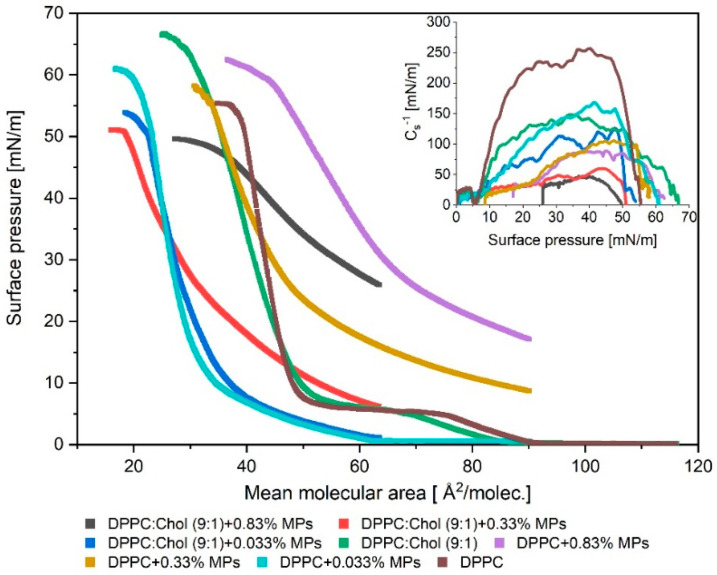
π–A isotherms of Langmuir monolayer for DPPC and DPPC:Chol (9:1) with different concentration of MPs; the inset graph presents surface compressional modulus *C_s_*^−1^ vs. surface pressure, π.

The π–A isotherm is an efficient technique to study the phase behavior of Langmuir monolayers and describe the thermodynamic interactions among the film’s components at a molecular level. Measurements of the π–A isotherms were carried out during the compression of the monolayer composed only of DPPC phospholipid particles and composed of DPPC and Chol particles in the presence of MPs. To form the mixed films, 32 µL of a mixture of MPs (of different concentrations, that is, 0.033%, 0.33% and 0.83%) and lipids (DPPC or DPPC:Chol) were spread over the subphase surface. The results obtained for the DPPC + MPs and DPPC:Chol (9:1) + MPs monolayers are shown in Figure 1. The isothermal π–A parameters values are given in Table 1.

The addition of Chol to the DPPC monolayer caused a slight shift in the π–A isotherm position toward lower mean molecular area values. The lift-off area of the DPPC monolayer was ca. 88 Å^2^/molec., while for DPPC:Chol (9:1) it was ca. 84 Å^2^/molec. The presence of Chol increased the stability of the phospholipid film, which collapsed at 67 mN/m while the DPPC monolayer reached collapse at ca. 55 mN/m. The addition of Chol to the DPPC + 0.033% MPs system did not significantly affect the π–A isotherm position. For the DPPC + 0.033% MPs and DPPC:Chol + 0.033% MPs, the A_lift-off_ values were similar and they reached ca. 61 Å^2^/molec. and ca. 64 Å^2^/molec., respectively. What is worth emphasizing is the fact that the mixed DPPC:Chol + 0.033% MPs monolayer collapsed at a lower surface pressure (ca. 54 mN/m) than the DPPC + 0.033% MPs monolayer (ca. 61 mN/m). According to literature reports [31,32,33], when the π–A isotherm obtained for the Chol monolayers is characterized by a large region with an area per molecule greater than 50 Å^2^/molec. and experiences further compression, a sudden increase in the surface pressure is observed until the collapse point at ca. 46 mN/m [34]. For this reason, it can be assumed that the addition of Chol to the mixed monolayer of DPPC and MPs will be conducive to lowering the collapse point. Both the DPPC + 0.033% MPs as well as the DPPC:Chol + 0.033% MPs monolayers reached areas much smaller than those only occupied by DPPC molecules under the compression process. In our previous work [27], we argued that this effect was due to the adsorption of DPPC molecules on MPs, which also contributes to reducing the concentration of phospholipids at the air–water interface. Now, we showed that the addition of Chol has no significant effect on DPPC + 0.033% MPs and DPPC:Chol (9:1) + 0.033% MPs systems.

The addition of Chol in the model lipid monolayer significantly impacted the position of the π–A isotherms for systems containing 0.33% or 0.83% concentrations of MPs. The π–A isotherms for these systems, DPPC:Chol (9:1) + 0.33% MPs and DPPC:Chol (9:1) + 0.83% MPs, were shifted towards a lower molecular area (A) with respect to the monolayer without Chol. Compared with the DPPC + MPs monolayers, the lift-off area values were greater than 64 Å^2^/molec. for both the DPPC:Chol (9:1) + 0.33%MPs and DPPC:Chol (9:1) + 0.83% MPs.

Moreover, mixed monolayers with Chol formed less stable lipid films because their collapse occurred at lower surface pressure values. A particularly highly condensed film was obtained for the DPPC:Chol (9:1) + 0.33% MPs system. We found that, for the considered systems, the addition of Chol allows one to obtain more compressed films. At 30 mN/m, the DPPC:Chol (9:1) + 0.33% MPs system reached a minimum molecular area ca. 28.4 Å^2^/molec., while for DPPC + 0.33% MPs, the minimum molecular area was ca. 44.8 Å^2^/molec. In this case, the addition of Chol caused a reduction in the area per molecule by 37% in comparison to the area per molecule value obtained for the DPPC + 0.33% MPs monolayers. A similar effect was found for the systems with 0.83% MPs, but for this film, the presence of Chol in the monolayer led to the reduction in area per molecule of ca. 12.5% (at 30 mN/m) in reference to the DPPC + 0.83% MPs system. The incorporation of MPs at a concentration of 0.33% MPs and 0.83% MPs and Chol resulted in a sudden increase in the surface pressure. The initial surface tension was ca. 8 mN/m for the DPPC:Chol (9:1) + 0.33% MPs and ca. 25 mN/m for the DPPC:Chol (9:1) + 0.83% MPs before the beginning of the monolayer compression. The increase in the initial surface pressure was greater the higher the concentration of MPs at the air–water interface. The change in the position and slope of the π–A isotherm may have resulted not only from interactions between the DPPC and MPs (we described this effect in our previous paper [27]) but also as a consequence of the presence of additional interactions between Chol and silica. As has been shown [24], the presence of nanoparticles in the Chol monolayer causes an increase in the surface pressure value with a lower degree of compression, and it reduces the value of the collapse pressure value. These observed effects may result in the formation of hydrogen bonds between the nondissociated silanol groups on the surface of the nanoparticles and the hydroxyl group of the Chol molecules. It is well known [35] that the Chol molecules form insoluble monolayers. Upon the compression of the monolayer, Chol molecules tend to orient themselves with the polycyclic hydrophobic part toward the air phase, whereas the hydroxyl group remains in the water phase. According to the literature [24], ellipsometry experiments have shown that nanoparticles penetrate into the Chol monolayer. The nanoparticles form partially hydrophobic complexes, but these complexes are not transferred back to the aqueous subphase as a result of the higher hydrophobicity of Chol. In consequence, the particles are trapped at the interface and simultaneously interfere with the monolayer compression because they induce steric hindrance.

The occurrence of this spatial obstruction causes higher values of surface pressure at areas per molecule to be recorded during compression for the film containing complexes than for pure Chol. The maximum packing of the mixed monolayer Chol with nanoparticles is achieved for a greater area per molecule compared to the pure Chol film. This effect also explains why the decrease in the collapse surface pressure can be observed for the Chol monolayer with nanoparticles.

It is clearly seen from Figure 1 and data tabulated in Table 1 that though the collapse pressures of the pure components (Chol and DPPC) were similar, the collapse pressures of the mixed monolayers varied with composition. Moreover, all of the mixed monolayers were characterized by only one collapse point. According to the phase rule, the isotherm of a monolayer consisting of the immiscible components will show two distinct collapse pressures corresponding to pure components [32]. Thus, it can be concluded that the DPPC, Chol and MPs were miscible at the air–water interface.

**Table 1:** Characteristic parameters of π–A isotherms: A_lift-off_—lift-off area of surface pressure; π_collapse_ —collapse pressure; A_collapse_—area corresponding to the monolayer collapse; max. *C_s_*^−1^—maximum value of the compression modulus.

	A_lift-off_ [Å^2^/molec.]	A_collapse_ [Å^2^/molec.]	π_collapse_ [mN/m]	$\max.C_{s}^{-1}$ [mN/m]
DPPC	88	35	55.3	254.1
Cholesterol *	52	38	41.0	693.0
DPPC:Chol (9:1)	84	25	67.1	144.1
DPPC + 0.033% MPs	61	18	60.9	162.6
DPPC:Chol(9:1) + 0.033% MPs	63	19	53.8	125.2
DPPC + 0.33% MPs	<90	31	58.2	104.7
DPPC:Chol (9:1) + 0.33% MPs	<64	18	50.8	60.2
DPPC + 0.83% MPs	<90	37	62.4	88.7
DPPC:Chol (9:1) + 0.83% MPs	<64	29	49.5	46.1

* Date from literature [34].

On the basis of the compression modulus values, *C_s_*^−1^, it can be concluded that the presence of cholesterol particles affects the elasticity of the monolayer. The compression modulus is defined as follows [31,36]:

$$C_{s}^{-1}=-A\cdot {\left(\frac{d\pi }{dA}\right)}_{T},$$

Davies and Rideal (1963) introduced the *C_s_*^−1^ parameter in order to describe the behavior of amphiphilic molecules at the air–water interface. The value of *C_s_*^−1^ allows us to have better insight into the physical state and/or packing changes in the molecules in the monolayer [37]. The established ranges of the *C_s_*^−1^ modulus correspond to certain physical states of the monolayer, i.e., the liquid-expanded (LE) and liquid-condensed (LC) states are characterized by the *C_s_*^−1^ values in the range of 12.5–50 mN/m and 50–250 mN/m, respectively. According to the literature [38], the *C_s_*^−1^ limit values for the solid state (LS) are 1000–2000 mN/m.

The inset (Figure 1) shows the variation in *C_s_*^−1^ during the film compression and as a function of surface pressure (π) obtained for the DPPC + MPs and DPPC:Chol + MPs monolayers spread on water. For the Chol monolayer spread over pure water, the *C_s_*^−1^ suddenly increases with the increase in the surface pressure to a maximum value of *C_s_*^−1^ above 500 mN/m, which indicates the formation of solid-like films [31,37,38]. This monolayer is characteristic of a very rigid structure. The monolayer consists of only DPPC molecules and also forms a film in the solid state, but in this case a maximum value of *C_s_*^−1^ occurs around 254 mN/m. The formation of a mixed DPPC:Chol monolayer leads to a significant decrease in the value of the elastic modulus and a reduction in the rigidity of the created film. For the DPPC:Chol (9:1) monolayers, the max. *C_s_*^−1^ value obtained was ca. 144 mN/m.

The addition of MPs to the phospholipid (Figure 1) or DPPC:Chol monolayer led to lower values of elasticity. This is consistent with other reports in the literature [24,27,39]. As mentioned earlier, the introduction of MPs is responsible for the appearance of a steric hindrance to the molecular packing and the subsequent decrease in the rigidity of the lipid monolayer. As is well known, the incorporation of Chol particles into the DPPC monolayer leads to a change in its elasticity. Chol molecules are characterized by high hydrophobicity, so they tend to distribute among the DPPC molecules to minimize the contact of the hydrophobic sterol ring with water. A tetracyclic fused ring along with a flexible hydrocarbon side chain helps to attract phospholipid acyl chains. As a result, an ordered structure is formed, which is strongly stabilized by the van der Waals cohesive interactions between the sterol rings of Chol molecules and the alkyl chains of DPPC [31,40]. Therefore, the presence of Chol molecules has a condensing effect on the DPPC and DPPC + MPs monolayers. These changes in the monolayer structure were clearly visible in the modulus of the elasticity values obtained for the systems: DPPC:Chol (9:1) + 0.033% MPs, DPPC:Chol (9:1) + 0.33% MPs and DPPC:Chol (9:1) + 0.83% MPs, where the introduction of Chol particles into the monolayer caused a reduction in the max. *C_s_*^−1^ by 37%, 38% and 48%, respectively. As shown in Figure 1, the incorporation of MPs or Chol particles into the DPPC film caused significant changes in the elasticity of the lipid monolayer. This can be seen from the compressibility curves shown in the inset of Figure 1 and the maximum values of *C_s_*^−1^ for the investigated mixed monolayers (Table 1). Based on these results, it was found that almost all the films studied formed liquid-condensed (LC) states because the max. *C_s_*^−1^ was in the range of 60–125 mN/m, and only the DPPC:Chol (9:1) + 0.83% MPs created the liquid-expanded (LE) state (max. *C_s_*^−1^ < 50 mN/m). Generally, one can observe that the addition of the Chol particles to the DPPC+MPs films caused the fluidizing effect in monolayers. Moreover, this fluidizing effect was stronger the greater the concentration of silica molecules in the DPPC:Chol + MPs system.

**Figure 2:**
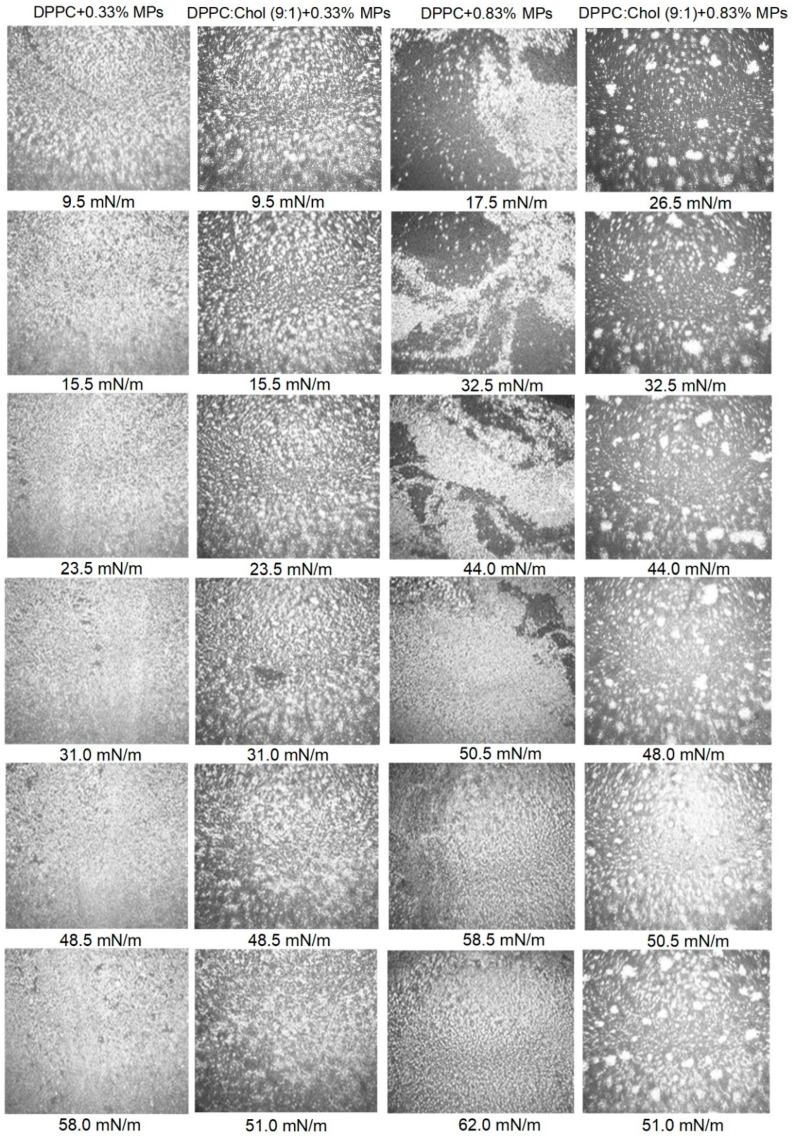
BAM images of DPPC + MPs and DPPC:Chol (9:1) + MPs at different surface pressures.

In order to obtain morphological information on the formed monolayers, we used the BAM technique. The BAM images for the analyzed systems are presented in Figure 2. The BAM images for the monolayers of the bare DPPC, bare DPPC:Chol (9:1) and bare MPs are included in the Supplementary Materials (Table S1) and in our previous work [27]. According to the literature [38], a Chol monolayer is characterized by a smooth and bright surface and is visualized without any domains or aggregates up to the collapse point. Chol forms a stable and condensed monolayer.

As shown [27], the addition of MPs to the surface of the DPPC monolayer resulted in the formation of a heterogeneous structure. The interactions between the MPs and phospholipid particles led to the formation of domains. The method of aggregate formation strongly depended on the concentration of MPs deposited on the lipid film. The addition of 0.33% of the MPs created domains that were regularly scattered over the entire surface, while the addition of 0.83% MPs favored the formation of large, irregularly shaped aggregates. The DPPC monolayer with deposited MPs gradually covering the surface with agglomerates formed the condensed liquid phase at a collapse surface pressure. The compression of the DPPC monolayer with deposited MPs led to a gradual covering of the surface with agglomerates and, as a result, the formation of the strongly condensed liquid phase, especially at the collapse surface pressure. The presence of Chol in the DPPC + MPs system caused the formation of monolayers with a more regular dispersion of domains at the interface. Guzmán et al. [31] also observed that the nanoparticles were more homogenously distributed along the monolayer and less aggregated with lipid monolayers containing Chol.

Moreover, the achieved BAM images also proved that Chol molecules have a tendency to distribute among the DPPC molecules, thus preventing the formation of any large aggregates, which is especially visible in the comparison of systems with 0.83% MPs with or without a contribution of Chol molecules. The obtained BAM images for the DPPC + MPs and DPPC:Chol + MPs systems indicated clear differences in the morphology of the monolayers, which also confirmed the existence of interactions between MPs and Chol molecules, leading to the formation of MPs + Chol complexes at the interface.

### 2.2. Relaxation Experiment

#### 2.2.1. Effect of Cholesterol Addition on the Stability of DPPC + MPs Monolayers

**Figure 3:**
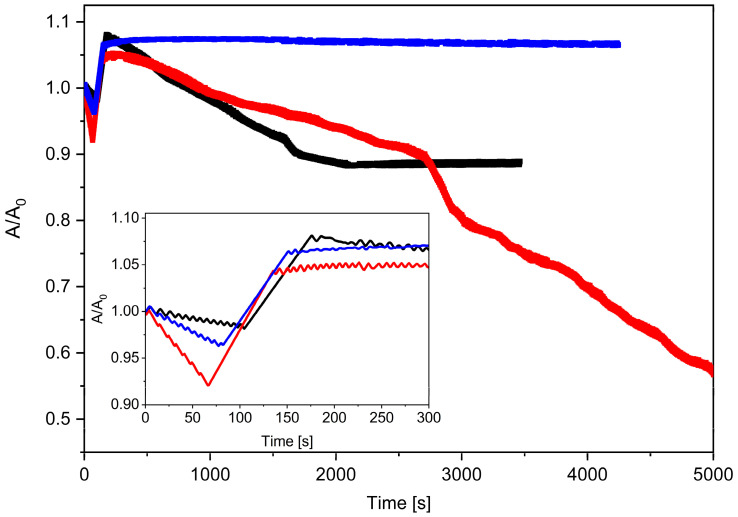
Relative area–time curves for DPPC + 2.5% MPs (20 µL) with different molar ratios of cholesterol (black line—DPPC + MPs; red line—DPPC:Chol (1:1) + MPs; blue line—DPPC:Chol (9:1) + MPs).

The investigated monolayers were compressed to 30 mN/m and then, after an injection of 20 µL of MPs, the change in the relative surface A/A_0_ vs. time was recorded (Figure 3). On the basis of the run of the relaxation curves, it was found that the stability of the monolayer depended on the amount of the Chol molecules incorporated into the binary film of the DPPC + 2.5% MPs (at the injected dispersion volume of 20 µL). The addition of a significant amount of Chol (ca. 50% mol.) clearly destabilized the DPPC + 2.5% MPs film, while about 10% mol. of Chol. allowed for the formation of a very stable monolayer.

Many studies indicated that the interactions of Chol molecules with other lipids play an important role in the formation of rafts in animal cell membranes. Most often, a narrow range of Chol molecules concentration (up to 50%) [41,42] is used in the lipid monolayer due to the limited solubility of Chol in the membrane. Above Chol solubility, monohydrate crystals or bilayer domains [24] are formed, which are generally considered as a symptom of membrane pathology. The concentration of Chol in the lipid membrane influences the permeability of the membrane to small polar and nonpolar molecules. Yue et al. [43] indicated that the Chol content remarkably influenced the fluidity of the DPPC monolayer. The increased Chol content led to a more orderly arrangement of lipid molecules and enhanced the packing of the hydrophobic portion of the lipid molecule. Thus, the fluidity of the DPPC monolayer was reduced and in consequence, the MPs encountered increased difficulty in penetrating the monolayer.

For each of the tested systems, one can observe the reduction in the A/A_0_ value at the beginning of the relaxation process and then an increase in the relative A/A_0_ value after ca. 50 s (Figure 3 inset). Interestingly, the observed effect of the decreased A/A_0_ value was more pronounced the greater the Chol content in the studied monolayers. Moreover, as in our previous work [27], in this case we also observed some cyclic changes in the A/A_0_ values for the investigated systems containing Chol molecules. The greatest tendency to stabilize of the DPPC + 2.5% MPs monolayer was shown by the system containing 10% mol. Of Chol. The relaxation curves of the DPPC:Chol (9:1) + 2.5% MPs were horizontal lines showing negligible film loss by dissolution or evaporation. A similar relaxation effect was shown for the Chol:DMPC system [44], where the small amount of Chol improved the lipid stability of the monolayer. On the other side, for monolayers with 50% mol., the dramatic drop in the A/A_0_ value indicated that the monolayer was slowly collapsing because there could have been three-dimensional structures forming during the relaxation process. In this case, the drop in its run led to a ca. 50% loss in area at a rate decreasing with time during the first 10 min.

**Figure 4:**
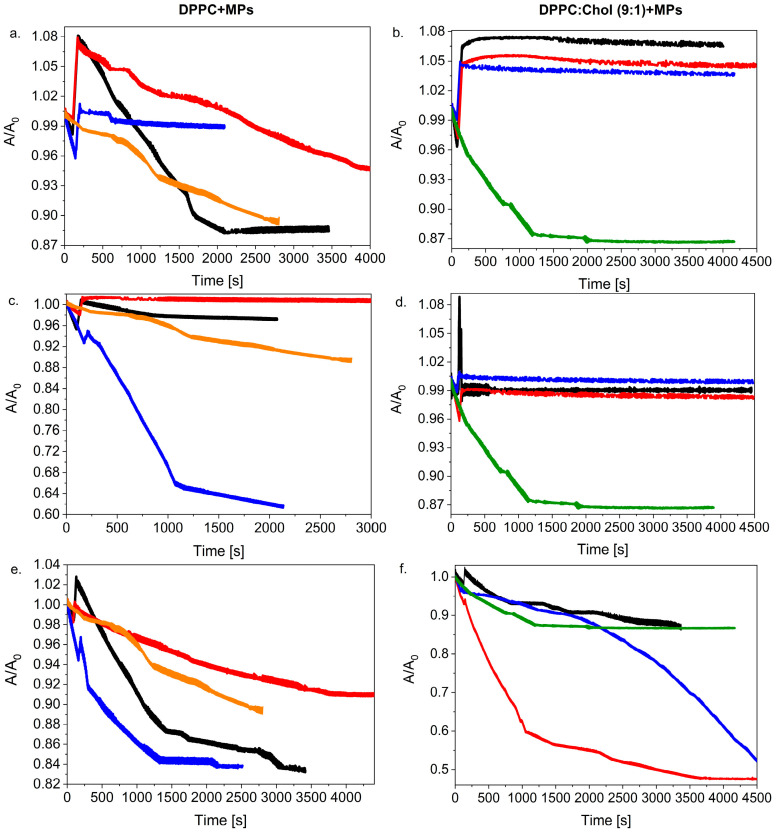
Relative area–time curves for various concentrations of silica particles (0.1%, 1.0% and 2.5%) injected on DPPC or DPPC:Chol (9:1) with volume of 20 µL (**a**,**b**), 10 µL (**c**,**d**) or 5 µL (**e**,**f**) (black line—MPs 2.5%; red line—MPs—1.0%; blue line—MPs—0.1%; orange line—DPPC (without MPs); green line—DPPC:Chol (9:1) (without MPs)).

Figure 4 compares the run of the relaxation curves for monolayers with or without Chol molecules. It is clearly visible that regardless of the concentration and volume of the MPs added to the system, the presence of Chol definitely influenced the run of the relaxation curves of the considered monolayers. One can conclude that for the systems with higher volumes of MPs (10 µL or 20 µL), the presence of Chol in the monolayer had an impact on the stabilization of the DPPC + MPs film.

In the case of spreading 20 µL of 1.0% and 2.5% suspended MPs on the DPPC:Chol (9:1) monolayer, even an increase in the absolute value of A/A_0_ was observed, which proves the incorporation of the MPs into the structure of the lipid film. These systems formed stable monolayers at the beginning of the relaxation process, in contrast to the DPPC monolayer, where the addition of 2.5% MPs caused the disintegration of the lipid film, and finally, the value of A/A_0_ stabilized at 0.88.

On the other hand, for 10 µL of the dispersed MPs (Figure 4c,d), the presence of Chol molecules had the greatest impact on the relaxation of the lipid film with the addition of MPs. The relaxation of the DPPC + MPs monolayer showed a great degree of disintegration of the lipid film and stabilized after a ca. 40% loss of area (after ca. 2000 s). However, the distribution of the same dispersion (10 µL of MPs) on the surface of the DPPC:Chol (9:1) monolayer completely changed the kinetics of the formed relaxation process; the film was stable and did not disintegrate.

In turn, the application of 5 µL of MPs solutions caused their smaller local distribution on the surface of the lipid film, which resulted in a change in the course of the relaxation curves. In this case, the presence of the Chol molecules in the lipid film did not help improve the stabilization of the monolayer with 0.1% dispersion of the MPs. We observed a really strong disintegration of the DPPC:Chol (9:1) + 1.0% MPs film, which consequently led to the loss of ca. 50% of the initial molecular area A_0_. The film disintegration process was also observed for the system with 0.1% MPs, but the kinetics were changed significantly. In the presence of Chol molecules in the mixed monolayer, the decrease in the A/A_0_ value occurred slowly at the beginning of the process (Figure 4f) in contrast with the DPPC + 0.1% MPs (Figure 4e). After ca. 2000 s, the A/A_0_ for DPPC:Chol (9:1) + 0.1% MPs drastically decreased, leading to a strong disintegration of the film (a surface reduction of more than 60% after ca. 5500 s). 

In general, it was observed that the presence of Chol in the monolayer favored the formation of more stable films involving. This effect also proved the fact that Chol and DPPC molecules form a more condensing monolayer. Chol molecules tend to distribute among the DPPC molecules in order to minimize the contact of the hydrophobic sterol ring with water. The strong interaction between the sterol chain and the alkyl chains of the DPPC molecules induced a high cohesion between the molecules at the interface and formed a stable film. The addition of MPs can improve this stabilization because there are new interactions between MPs and Chol or DPPC molecules. This allows one to assume that the process induced by the presence of MPs consists of a rearrangement of the mixed monolayers in a three-dimensional structure composed of lipid–nanoparticle complexes [38] that allows one to obtain a more stable monolayer.

#### 2.2.2. Influence of MPs Distribution on the Stability of the Lipid Monolayer

**Figure 5:**
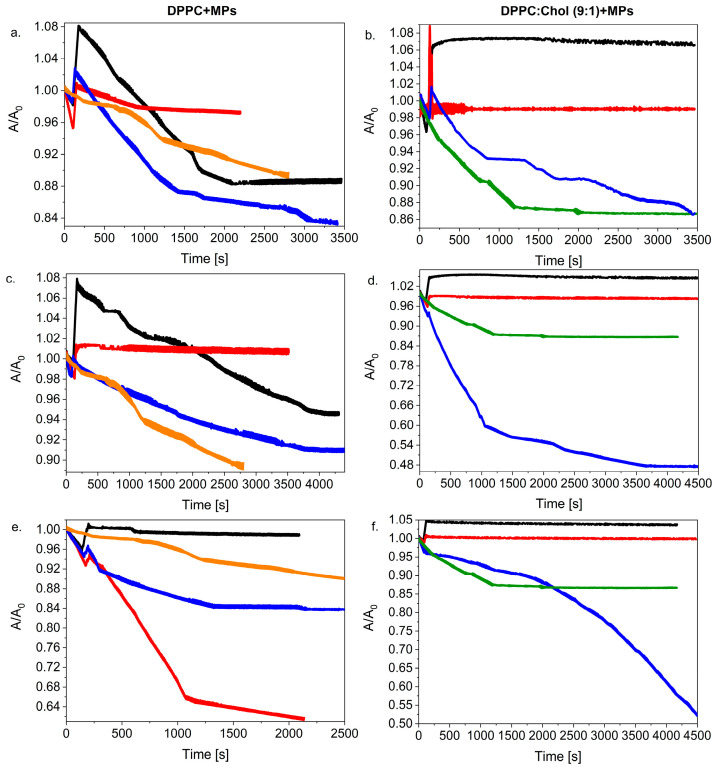
Relative area–time curves for various volumes of silica particles (5, 10 and 20 µL) injected on DPPC or DPPC:Chol (9:1) with concentration of 2.5% (**a**,**b**), 1.0% (**c**,**d**) or 0.1% (**e**,**f**) (black line—20 µL MPs; red line—10 µL MPs; blue line—5 µL MPs; orange line—DPPC (without MPs); green line—DPPC:Chol (9:1) (without MPs)).

In the next stage of our research, we investigated how changing the volume of the dispersed silica influences the DPPC and the DPPC:Chol monolayer stability. In Figure 5, we compared these two systems taking into account different MP concentrations: 0.1%, 1.0% and 2.5% and various volumes (5 µL, 10 µL and 20 µL).

As mentioned in our previous paper [27] and according to the literature [24,33,45], we expected that the dosing method and the distribution of the MPs on the surface of the lipid films would impact the reduction in the surface tension of the analyzed model membranes. Moreover, it could be assumed that a greater volume and higher concentration of MPs increased the possibility of MPs interacting with each other and could finally favor formulation of agglomerates. We showed [27] that no simple correlation was found between the dispersion volume added to the DPPC monolayer and its stability. However, generally, good stability films were formed when the dispersion particles were injected with a higher concentration of MPs as 1.0% or 2.5% (Figure 5a,c). Now, if we compare two systems, e.g., the DPPC + MPs and the DPPC:Chol + MPs, we can consider that the presence of the Chol in the lipid films leads to the formation of a more stable system. The effect of the presence of Chol was especially visible in the case of the high local distribution when injecting the highest volume (20 µL) and concentration of silica particles. On the other hand, one can observe the opposite effect for systems with a low dispersion of MPs on the lipid membrane surface. The addition of 5 µL of MPs with a concentration of 0.1% to DPPC:Chol film caused a strong degradation of the lipid film even though the value of A/A_0_ slowly decreased at the beginning. Consequently, 50% of the initial area was lost after ca. 4500 s (Figure 5f). Moreover, an increase in the concentration of the MPs led to a change in the direction of the relaxation curves. For both the DPPC:Chol + 1.0% MPs and the DPPC:Chol + 2.5% MPs systems, a sudden decrease in the A/A_0_ value and then the stabilization of the relaxation curve was clearly visible (Figure 5b,d).

## 3. Materials and Methods

### 3.1. Materials

1,2-dipalmitoilo-*sn*-glicero-3-fosfocholina (DPPC) (≥99%) and cholesterol (Chol) (≥99%) were purchased from Sigma-Aldrich (Wrocław, Poland). High purity chloroform for spectroscopy (Uvasol), isopropanol (≥99.8%) and acetone (≥99.8%) were purchased from Merck (Wrocław, Poland). The research material used was commercially modified silicate (CLOISITE SE 3000, BYK, Wesel, Germany) based on natural bentonite with an average particle size <10 µm (MPs). The analyzed material (silica particles) was stored in a special desiccator, which prevented the access of bacteria to the surface of the silica particles. Detailed characteristics of the microparticles were presented in the Supplementary Materials (Figure S1) and in our previous work [27].

### 3.2. Isotherm π–A Experiments

**Figure 6:**
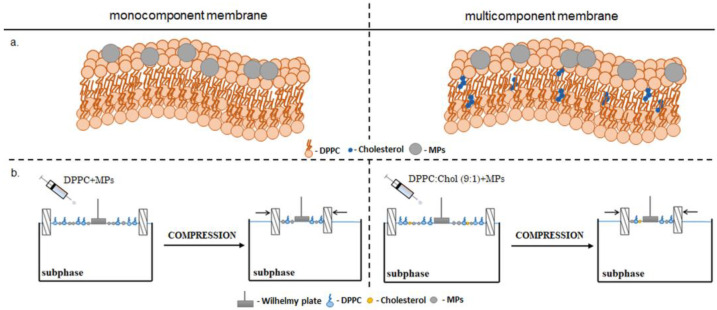
The idea of the Langmuir monolayer method: (**a**) schematic drawing of the model membranes, (**b**) schematic idea of isotherm π–A experiments.

A DPPC solution (1 mg/mL), a Chol solution (0.53 mg/mL) and MPs dispersion (0.1% *w/w*, 1.0% *w/w*, 2.5% *w/w*) were prepared by dissolving the solids in chloroform. Prepared dispersions were placed in an ultrasonic cleaner for about 30 min before the measurements were carried out. The DPPC:Chol (9:1) system was formed by mixing DPPC and Chol in a 9:1 molar ratio. Similarly, the DPPC:Chol (1:1) system was prepared. To obtain mixtures of the substances used for the formation of the monolayers, MP dispersions were mixed with lipid mixtures (DPPC or DPPC + Chol) in a volume ratio (1:2). Therefore, we prepared mixtures of 50 µL of MPs (the initial concentration with 0.1% *w/w*, 1.0% *w/w* and 2.5% *w/w* of MPs) and 100 µL of lipids. Then, 32 µL (containing 0.033%, 0.33% and 0.83% MPs, respectively) of this mixture was evenly applied with a microsyringe (Hamilton, Gdynia, Poland) to the surface of the subphase placed in the Langmuir trough (KSV Nima, Espoo, Finland) with a total area of 273 cm^2^. Ultrapure water (18.2 MΩ·cm, 71.98 ± 0.01 mN/m) was used as the subphase. The schematic process of forming mono- and multicomponent membranes was shown in Figure 6.

After the chloroform was evaporated from the mixture (15 min), the layer was compressed by the symmetric movement of two hydrophilic barriers made of Delrin polymer at a constant speed of 10 mm/min (3.75 cm^2^/min). A platinum Wilhelmy plate (instrument accuracy 0.01 mN/m) was used to measure the surface pressure. Before each measurement, the trough and barriers were cleaned with isopropanol, acetone and ultrapure water to obtain a surface pressure value for the pure subphase below 0.2 mN/m at maximum compression. During the compression of the monolayer with a computer-controlled Langmuir balance (KSV Nima, Finland), changes in the surface pressure (measured using a surface tension reduction measure, that is, a two-dimensional analogue of pressure) were recorded as a function of the surface area available for each molecule on the surface of the water subphase, that is, the surface pressure isotherm (π) − area (A). During the measurement, the temperature was kept constant by a Julabo F12 thermostat. From the π–A isotherm, the lift-off area (A_lift-off_) was determined as the molecular area, at which the surface pressure increases above 0 mN/m. Each experiment was repeated in triplicate to ensure the repeatability of the curves of π–A up to ± 2 Å^2^ at a constant temperature of 22.0 ± 0.1 °C.

### 3.3. Brewster Angle Microscopy (BAM)

The Langmuir balance was equipped with a Brewster angle microscope (MicroBAM, KSV Nima, Finland). The BAM images were recorded during the monolayer compression, which allowed us to visualize and monitor the structural changes in the surface membrane caused by the presence of MPs.

### 3.4. Relaxation Experiments

**Figure 7:**
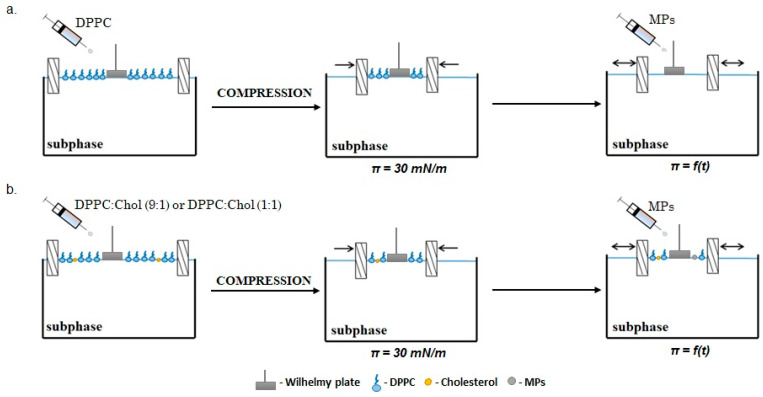
The schematic idea of relaxation experiments: (**a**) monocomponent membrane, (**b**) multicomponent membrane.

Relaxation experiments were performed to determine the stability of the monolayers. For this purpose, lipid solutions (DPPC, DPPC:Chol (1:1) and DPPC:Chol (9:1)) were first evenly distributed over the water subphase. After evaporating the solvent (15 min), the monolayer (with a constant speed of 10 mm/min) was compressed to reach a surface pressure of 30 mN/m. Next, the MP dispersions (with concentrations of 0.1% *w/w*, 1.0% *w/w* or 2.5% *w/w*) were injected onto the surface of the earlier-formed lipids’ monolayer (DPPC or DPPC:Chol) and then the relative surface area change (A/A_0_) was recorded over time (t). The MP dispersions were placed in an ultrasonic cleaner for about 30 min before the relaxation measurements were carried out. Each experiment was repeated in triplicate to ensure the repeatability of the curves up to ± 2 Å^2^ at a constant temperature of 22.0 ± 0.1 °C. The schematic course of the relaxation experiment for mono- and multicomponent membranes was shown in Figure 7.

## 4. Conclusions

On the basis of the presented research results, it can be concluded that the presence of Chol in the DPPC monolayer led to the formation of a more condensed and densely packed film than the monolayer created by only lipid molecules. As expected, changes in the structure of the molecular film impacted the permeability of the monolayer as well as the interactions between hydrophobic MPs with DPPC and Chol. The presence of Chol in the monolayer promoted the formation of MP–lipid complexes and thus more strongly supported the incorporation of MPs into the model phospholipid membrane.

The weight content of Chol in the lipid monolayer on the surface of which the dispersed MPs were applied determined the stability of the resulting surface film. A slight weight fraction of Chol (i.e., DPPC:Chol (9:1)) improved the stability of the surface film, which may have been due to the incorporation of solid particles into the monolayer. On the other hand, it was also shown that too high of a proportion of Chol in the DPPC monolayer (ca. 50%) led to its destabilization. Our experiments proved that the addition of Chol to the system that mimics biological model membranes affects the order and fluidity of the membrane, which play an important role in the proper functioning of the living cells.

## Data Availability

Not applicable.

## Supplementary Materials

The supporting information can be downloaded at: https://www.mdpi.com/article/10.3390/ijms232112822/s1.
