# Supplementary material for: Effect of Silica Microparticles on Interactions in Mono- and Multicomponent Membranes
Source: Int J Mol Sci. 2022 Oct 24;23(21):12822. doi: 10.3390/ijms232112822 (PMC9654498; doi:10.3390/ijms232112822)
Supplement: Supplementary file 1 [file ijms-23-12822-s001.zip › ijms-1906335-supplementary.pdf]

# SUPPLEMENTARY MATERIALS

## Characterization of the microparticles (MPs):

The dispersive properties of MPs were measured with a Zetasizer Nano ZS aparatus (Malvern Instruments Ltd., Worcester, UK), which enabled the measurement of particle sizes using the non-invasive backscattering method (NIBS). The sample (0.01 g) was dispersed in 25 mL of propan-2-ol, sonicated for 15 min, and then placed in a cuvette for analysis (Fig. S1a).

The surface morphologies of tested MPs were investigated by scanning electron microscopy (SEM, Tescan Vega 5135). The materials were sputter-coated with a thin layer of carbon for allowing the SEM visualization. The images were taken by applying the electron accelerating voltage of 15.0 kV. The SEM of the sample confirmed the presence of particles with small diameter (corresponding to those indicated in the particle size distribution), oval shape and high heterogeneity (Fig. S1b).

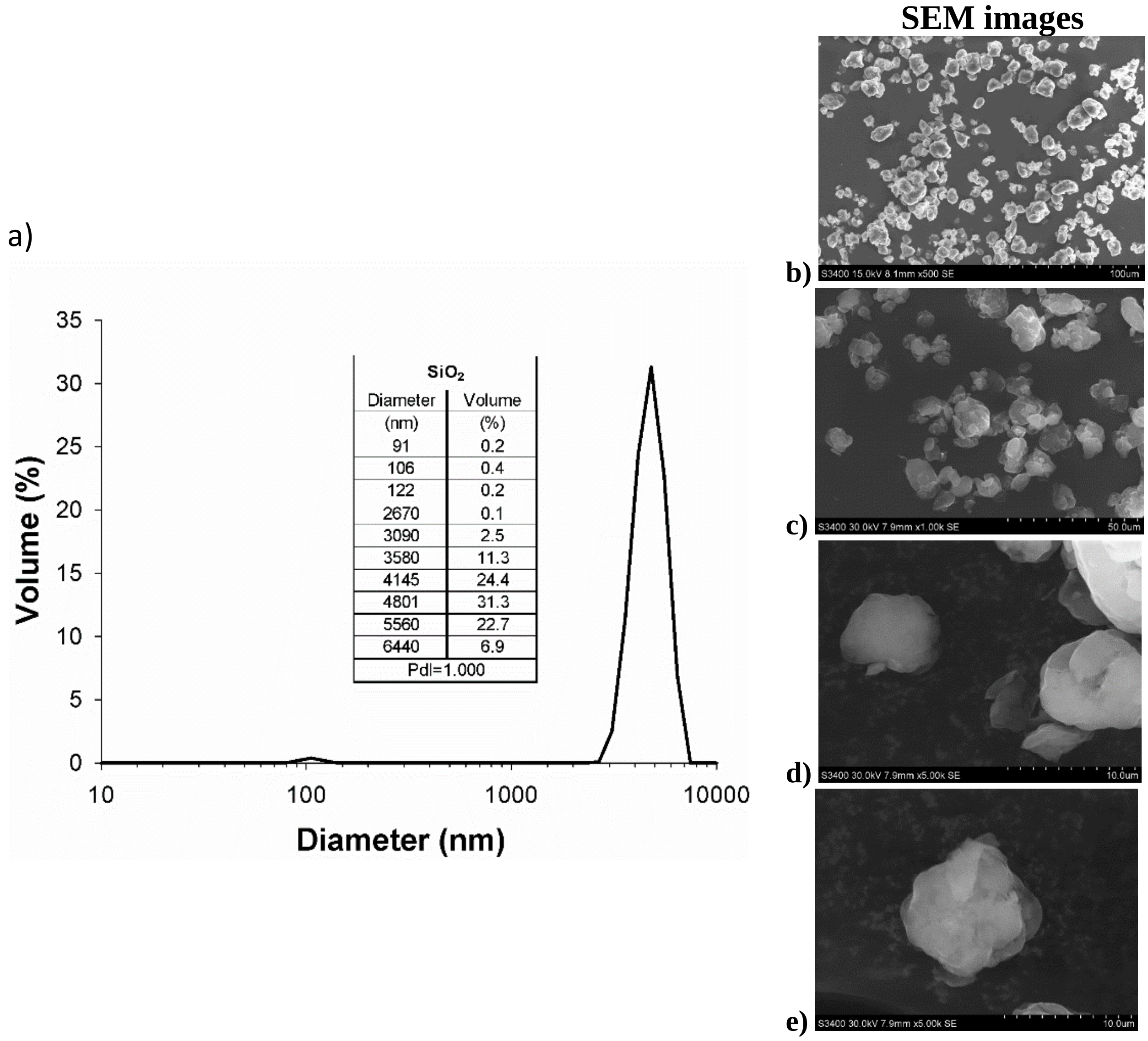


| Diameter (nm) | Volume (%) |
|---|---|
| 91 | 0.2 |
| 106 | 0.4 |
| 122 | 0.2 |
| 2670 | 0.1 |
| 3090 | 2.5 |
| 3580 | 11.3 |
| 4145 | 24.4 |
| 4801 | 31.3 |
| 5560 | 22.7 |
| 6440 | 6.9 |
| PdI=1.000 | |

**Fig. S1.** a) Particle size distribution of MPs.
Scanning electron microscope (SEM) images of tested MPs: b) x500 ; c) x1000; d) x5000 ; e) x5000.

**Table S1.** BAM images for bare monolayers of DPPC, DPPC:Chol (9:1) or monolayers of MPs with different concentrations.

| DPPC | DPPC:Chol (9:1) | MPs 2.5% | MPs 1.0% | MPs 0.1% |
|---|---|---|---|---|
| 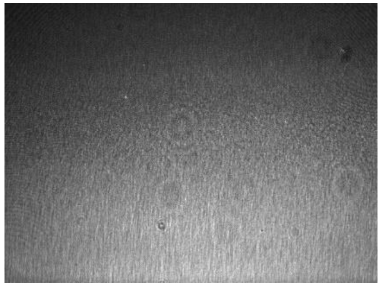 π= 5 mN/m | 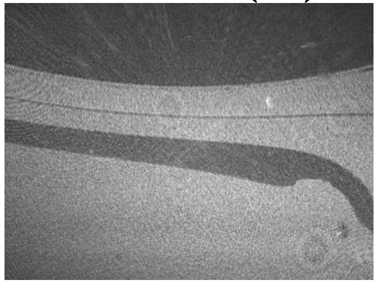 π= 0.5 mN/m | 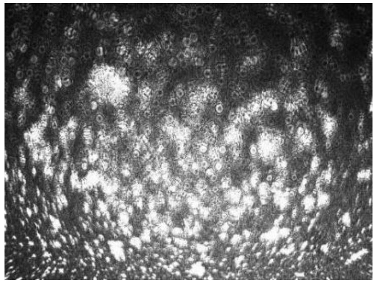 π=27 mN/m | 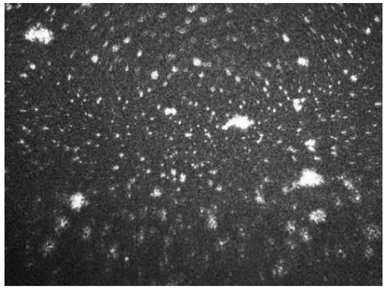 π=20 mN/m | 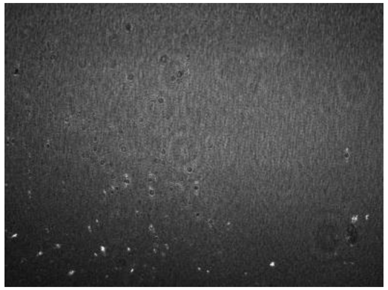 π=1.5 mN/m |
| 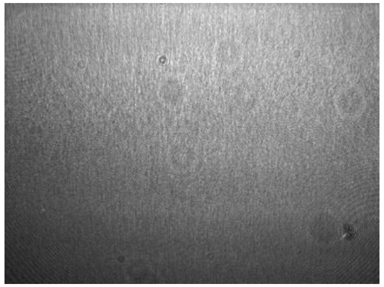 π=10 mN/m | 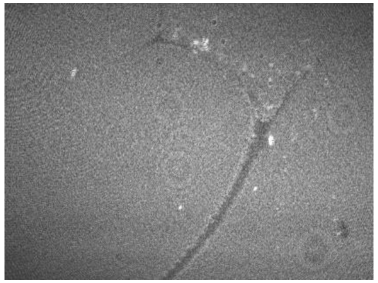 π=5 mN/m | 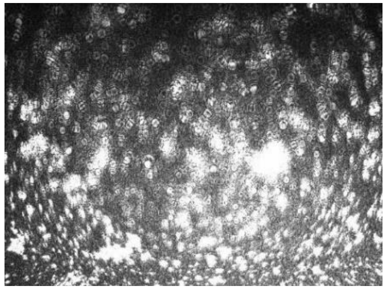 π=35 mN/m | 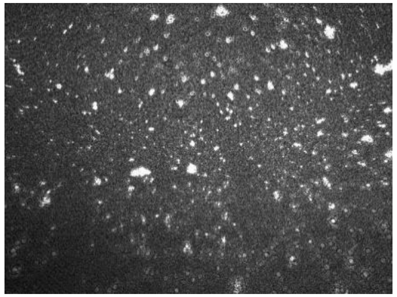 π=30 mN/m | 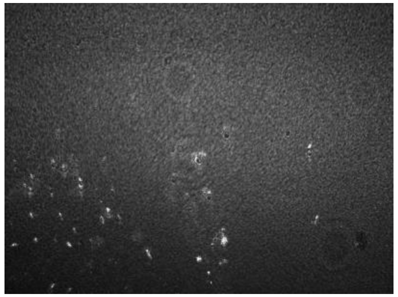 π=3.5 mN/m |
| 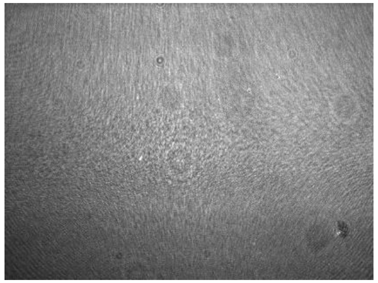 π=20 mN/m | 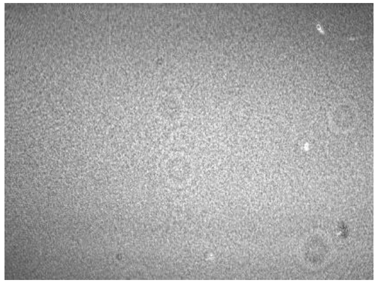 π=10 mN/m | 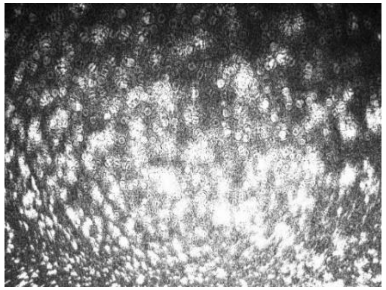 π=40 mN/m | 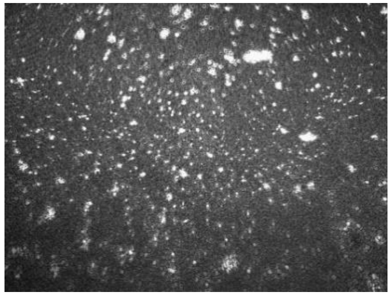 π=35 mN/m | 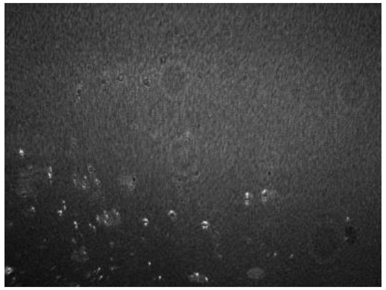 π=4.5 mN/m |

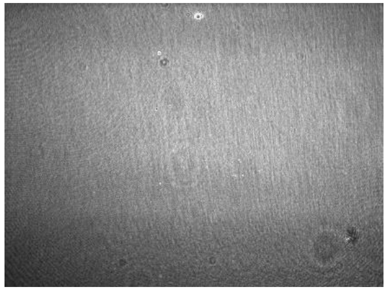

$\pi=35$ mN/m

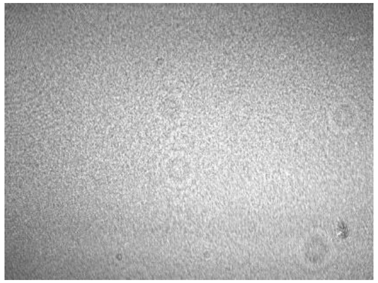

$\pi=25$ mN/m

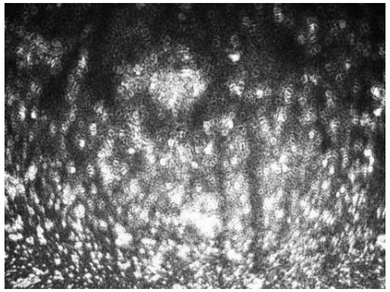

$\pi=45$ mN/m

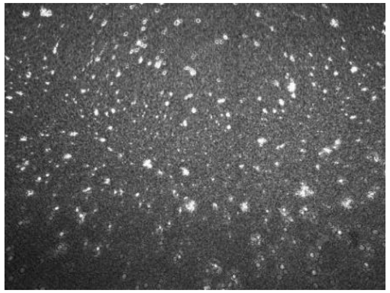

$\pi=40$ mN/m

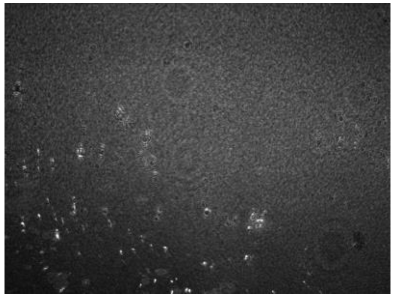

$\pi=6.0$ mN/m

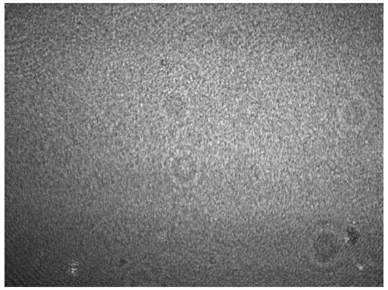

$\pi=45$ mN/m

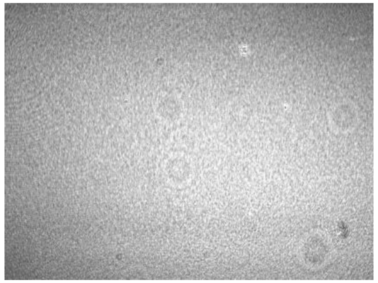

$\pi=45$ mN/m

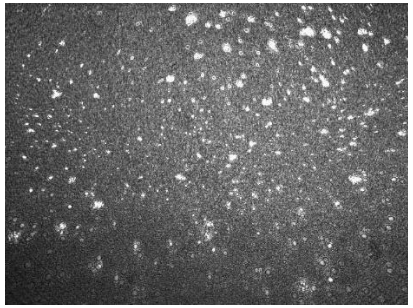

$\pi=45$ mN/m

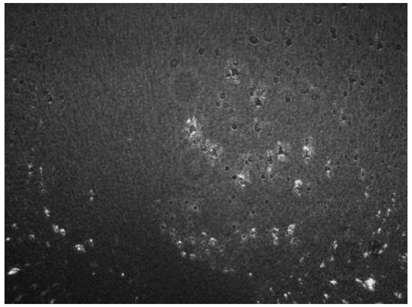

$\pi=8.0$ mN/m

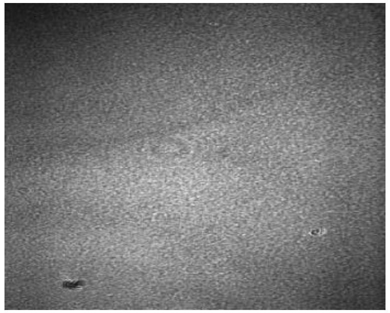

$\pi=54$ mN/m

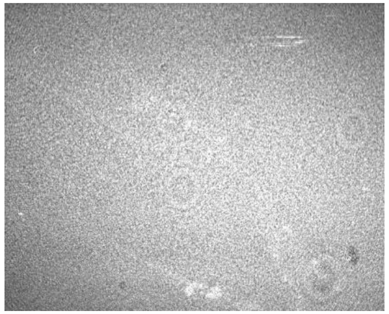

$\pi=54$ mN/m
